# Free Medical Schools

**Published:** 1852-01

**Authors:** 


### ARTICLE II.

### FREE MEDICAL SCHOOLS.

The Western Medico-Chirurgical Journal, which has heretofore been much opposed to the doctrine of Free Medical Schools, has come out in their favor. We suppose the school at Keokuk, for which this Journal is the organ, is preparing to apply to the State Legislature of Iowa for an endowment. If so, we hope the people of that State, and especially the intelligent members of our profession, (a large number of whom are subscribers to our Journal, for whose information we make this notice,) will see to it that when the Legislature endows an institution of the kind, it shall be one in which the whole profession of the State will have an interest, and not allow an endowment to go into private hands.

When Iowa, or any other State, has within its borders a city of sufficient size to afford the adequate means for medical instruction, we shall rejoice to see her properly and munificently endow and liberally sustain a free orthodox Medical School. Such an endowment should be accompanied by a provision requiring the chairs of the school endowed to be filled by concours or public trials.

